# Effects of Combinations of Dietary Vitamin C and Acetylsalicylic Acid on Growth Performance, Carcass Traits and, Serum and Immune Response Parameters in Broilers

**DOI:** 10.3390/ani14040649

**Published:** 2024-02-18

**Authors:** Giulia Ferronato, Masoomeh Tavakoli, Mehrdad Bouyeh, Alireza Seidavi, Lourdes Suárez Ramírez, Aldo Prandini

**Affiliations:** 1 Department of Civil Engineering, Architecture, Environment, Land Planning and Mathematics (DICATAM), Università degli Studi di Brescia, 25121 Brescia, Italy; 2 Department of Animal Science, Rasht Branch, Islamic Azad University, Rasht 41335-3516, Iran; mtavakoli23757@yahoo.com (M.T.); booyeh@iaurasht.ac.ir (M.B.); 3 Department of Animal Pathology, Animal Production, Bromatology and Food Technology, Veterinary Faculty, University of Las Palmas de Gran Canaria, 35412 Arucas, Spain; lourdes.suarez@ulpgc.es; 4 Department of Animal Sciences, Food and Nutrition (DIANA), Università Cattolica Sacro Cuore, 29122 Piacenza, Italy; aldo.prandini@unicatt.it

**Keywords:** poultry, ascorbic acid, acetylsalicylic acid, growth performance, carcass characteristics

## Abstract

**Simple Summary:**

Chicken meat has become increasingly popular as a protein source due to its favorable protein content and balanced amino acid profile. Advances in genetics and feeding practices have led to rapid growth in commercial broiler production to meet consumer demands. As consumer preferences shift toward healthier food options, there is a need to enhance meat production and quality in the poultry industry. Compounds with antioxidant properties, such as vitamin C (VC) and acetylsalicylic acid (ASA), have been tested due to their antioxidant and anti-inflammatory properties. Studies have shown positive effects of both in growth performance and immune functions. While individual studies have explored the effects of vitamin C and ASA supplementation in broilers, few have investigated their combined effects under non-stressful conditions. This study aimed to fill this gap and revealed significant effects of ASA and VC combined supplementation, with improvements noted in growth performance, carcass composition and immune response and cecal coli count. However, creatine kinase levels increased with ASA and VC intake. These findings highlight the complex interactions between ASA and VC supplementation in broilers. Further investigation is warranted to explore optimal dosages and potential effects in non-stressful conditions.

**Abstract:**

This study aimed to investigate the combined effects of ascorbic acid (VC) and acetylsalicylic acid (ASA) on broiler health and performance. A total of 200 Ross 308 male broilers were divided into five groups, each receiving different dietary combinations of ASA and VC (ASA: 50 or 100 mg/kg; VC: 200 or 400 mg/kg). The 42-day trial assessed parameters such as feed intake, average daily gain (ADG), feed conversion ratio (FCR), carcass characteristics, serum parameters, immune response and cecal microbial flora. The results indicate significant treatment effects on feed intake and growth performance, with a higher feed intake of ADG and FCR in treatment groups (*p* < 0.05). Serum lipid parameters were unaffected, but creatine kinase increased with ASA and VC intake (*p* < 0.05). Changes in sheep red blood cell titers and influenza antibodies were noted (*p* < 0.05). The combination of ASA and VC positively influenced carcass traits, reducing abdominal fat and altering the ratio of immune response organs to body weight (*p* < 0.05). Additionally, the cecal *E. coli* count decreased with treatment (*p* < 0.05). This study underscores the intricate interactions between ASA and VC supplementation, growth performance and carcass composition and immune response in broilers. Further research is warranted to explore dosage nuances and variations under specific stress conditions.

## 1. Introduction

In recent years, chicken meat has gained extensive use as a protein source, replacing red meat in numerous countries. Its marketability is attributed to its favorable protein content and well-balanced amino acid profile [1]. Additionally, advances in genetics and effective feeding practices have facilitated the rapid growth of commercial broilers to meet human demands, consequently boosting broiler production [2]. Contemporaneously, there has been a heightened focus on the quality of consumed food. Developed countries have shown interest toward maintaining health through the consumption of nutritious and healthful products, coupled with lifestyle modifications. This has prompted a desire for a more integrated approach to nutrition. The increasing awareness among consumers about health and the wealth of information available regarding the advantages of various diets and their impact on human well-being have driven the demand for functional food [3]. As quality and health become increasingly crucial factors influencing consumer preferences in food selection [4], and as consumers seek out healthy and safe food options, specific compounds should be utilized as antioxidants to improve meat production and enhance the overall quality of poultry meat within the industry.

Vitamin C and acetylsalicylic acid are two compounds that have beneficial antioxidant properties [5,6,7].

The role of vitamins and their uptake through feed are fundamental and essential for the survival and growth of living organisms [8]. Ascorbic acid, or vitamin C (VC), is one of the essential vitamins, with high antioxidant potential in biological systems due to its capacity to act against the lipid peroxidation of cell membranes and eliminate peroxyl radicals [8,9]. Moreover, vitamin C can enhance resistance to pathogens and improve the immune system, thus resulting in a better growth performance [10]. Birds have the ability to synthesize VC, but this ability is insufficient under stress conditions such as high or low temperature, relative humidity, a high productive rate and parasite infestation [11,12].

In the case of broiler chickens, several studies have shown a positive effect of vitamin C feed supplementation. Mirzapor et al. [13] noticed that the use of vitamin C significantly increased individuals’ weight during both the starter and grower phase. Attia et al. [14] stated that the use of vitamin C in the diet of broilers led to an increase in blood total protein, albumin, globulin, glucose and alkaline phosphate. Jahejo et al. [15] showed that the use of vitamin C supplements in broilers significantly improved white blood cells and red blood cells and thus improved the animal’s immune system, which was also reported in Angani et al.’s study [16]. Research has also established a reduction effect on the cecal coliforms count in association with an increase in lactobacilli forms [17]. On the other hand, broilers are prone to storing large amounts of fat in the body due to their genetic characteristics [18]. There are conflicting assumptions about the effects of antioxidants on serum cholesterol levels, although very few experiments have been performed on poultry in this regard. Zahagari et al. [19] showed that while vitamins, especially vitamin C, had no significant effect on total serum cholesterol levels, HDL cholesterol levels decreased and LDL cholesterol levels increased.

Acetylsalicylic acid (ASA), or aspirin, is a non-steroidal anti-inflammatory drug that inhibits the formation of prostaglandins by inhibiting the cyclooxygenase enzyme. This enzyme contains Cox-2, which is responsible for stimulating inflammatory responses and the formation of prostaglandins and therefore free radicals [20]. When this enzyme is inhibited, the acid acts as an antioxidant and protects the body from damage by free radicals [21]. Furthermore, this acid plays a key role in lowering cholesterol and triglycerides in blood, meat and eggs and improving immune function and antioxidant enzymes. Thus, its activity results in enhanced growth performance and feed utilization [5]. The use of acetylsalicylic acid as a feed additive in broilers has been tested mainly to reduce heat stress effects and growth performance [5,21,22,23], and the effect on growth varies with the dose. Positive effects were recorded when using the acid at levels higher than 0.30% [24], but a but negative one was noted with doses of 0.60% and 0.90% [25]. No effects were detected on carcass traits in Japanese growing quails [26], while carcass quality was improved in terms of pH and defects [27,28]. Moreover, the ratio of immune organs (thymus, spleen, bursa) to body weight was not affected by heat stress when acetylsalicylic acid was used. At hematological levels, ASA lowered cholesterol and triglycerides [29], but different results were found for other parameters [5,21,30]. ASA, with its ability to effectively inhibit free radicals, may solve the problems related to intestinal disorders and improve functional characteristics [31].

Several studies have examined the effects of using vitamin C and ASA as feed additives in broiler farming. However, these studies mainly focused on specific stress conditions and did not consider the combination of the two compounds. The purpose of this study was therefore to investigate the effects of the combined use of different doses of ASA and vitamin C on broiler chickens raised under non-stressful conditions. Specifically, the aim was to assess whether there was a combined effect of the two compounds at different dosages on growth performance, carcass characteristics, the gastrointestinal tract, blood parameters, the immune system and cecal microbial flora.

## 2. Materials and Methods

The experimental protocol was authorized by the Institutional Animal Care and Ethics Committee of Islamic Azad University (Rasht Branch, Rasht, Iran). The study was performed in a commercial broiler farm.

### 2.1. Experimental Design

A total of 200 one-day-old Ross 308 male broiler chickens (Aviagen, Newbridge, Scotland, UK) were purchased from a commercial farm and enrolled into a 42-day trial. The birds were randomly allocated into 5 treatment groups, each of which was assigned to 4 replicate floor pens with 10 birds per replicate.

The chickens were raised in cages measuring 1 m × 1 m and equipped with cellulose roll bedding. Uniform environmental conditions were maintained across all groups; the breeding hall temperature was set at 33 °C initially, gradually decreased to 23 °C by the 18th day of breeding and maintained at this level until the conclusion of the study. Throughout the entire duration, a consistent humidity level of 70% was maintained. Unrestricted access to water and food was ensured throughout the trial period.

In addition, birds were vaccinated against infectious bronchitis (at 10 days of age), Newcastle disease (at 4, 21 and 35 days of age) and infectious Bursal disease (at 12 days of age). The basal diet was the same for all 5 treatment groups. Particularly, a three-phase feeding strategy was used considering a starter diet (from 1 to 10 days of life), alongside a grower diet (from 11 to 24 days of life) and a finisher diet (from 25 to 42 days of life) (Table 1). The diet composition and requirements were assessed considering the Ross 308 manual recommendations [32].

Particularly, five experimental diets with different levels of ascorbic acid and acetylsalicylic acid (Iran Daru Pharmaceutical Co., Mashhad, Iran) were prepared:

- Treatment 1: control group (CTR) with basal diet;
- Treatment 2: basal diet plus 200 mg of vitamin C plus and 50 mg of acetylsalicylic acid (VC200-ASA50);
- Treatment 3: basal diet plus 400 mg of vitamin C plus and 50 mg of acetylsalicylic acid (VC400-ASA50);
- Treatment 4: basal diet plus 200 mg of vitamin C plus and 100 mg of acetylsalicylic acid (VC200-ASA100);
- Treatment 5: basal diet plus 400 mg of vitamin C plus and 100 mg of acetylsalicylic acid (VC400-ASA100).

Furthermore, to test the humoral immune response, all the subjects in each treatment group received vaccinations against infectious bronchitis at 10 days of age, Newcastle disease at 4, 21 and 35 days of age and infectious bursal disease at 12 days of age. All vaccines were sourced from the Razi Vaccine and Serum Institute (Karaj, Iran).

To investigate humoral immunity, broiler chickens were immunized against sheep red blood cells (SRBCs) according to the method of Lerner [33]. To prepare an SRBC suspension, three sheep were used for blood drawing and the blood was poured into a glass containing EDTA. The globules were washed three times in phosphate-buffered saline (PBS), and at the end of this, a suspension of 2% SRBCs was prepared in PBS. SRBC injection was performed at 28 and 36 days of age for two birds in each pen, injecting 0.1cc of the solution to the wing vein.

### 2.2. Growth Performance, Feed Intake and European Production Index

Body weight (BW) measurements were recorded on the first day and at the conclusion of each phase for every replicate within each treatment. Feed intake was recorded as the variance between the feed provided at the beginning of each period and the amount remaining at the end of each period. Additionally, the feed conversion ratio (FCR) was computed by dividing the feed consumption ratio by the weight gain for the respective individual period and the entire trial duration [34].

Moreover, the European Production Index (EPI) was calculated according to Equation (1) [35]:

(1) $$European\;Production\;Index=\frac{mean\;live\;weight\left(g\right)\times durability\left(\%\right)}{FCR\times averageage(d)}\times 100$$

Equation (1). European Production Index (EPI) formula.

### 2.3. Serum Parameters and Carcass Traits

At the end of the trial, 2 birds from each pen were randomly selected to measure blood parameters, and 5 mL of blood was taken before starvation from the wing vein. Blood serum was separated by centrifugation 5000× *g* rpm for 3 min (Eppendorf 5702, Hamburg, Germany) and stored at −20 °C until analysis. The serum samples were analyzed to measure the levels of glucose, triglycerides, cholesterol, total protein, albumin, globulin, creatine kinase, lactate dehydrogenase, high-density lipoprotein (HDL), very-low-density lipoprotein (VLDL), low-density lipoprotein (LDL), alanine transaminase and alkaline phosphatase. These parameters were measured using commercial kits of Pars Azmoon Corp. and the autoanalyzer apparatus (Hitachi 917, Tokyo, Japan) from Golrokh et al. [36].

Blood samples were also analyzed to count the total number of white blood cells and determine their differential counts. The determination of blood cell count was performed via cell staining, cell isolation and counting under an optical microscope [37].

After two hours of starvation, 2 birds from each replicate of each treatment were slaughtered. Then, the weights of the featherless carcass, full and empty body, breast, thigh and wing, as well as the digestive organs (pancreas, heart, gizzard, liver, ventricular fat, duodenum, jejunum and ileum), were measured [37]. The weights of the spleen, bursa of Fabricius and thymus were measured using a digital scale (A&D GF-300 digital scale balance (310 gr × 0.001 gr, A&D Weighing Design and Manufacture, San Jose, CA, USA) with an accuracy of 0.01 g.

### 2.4. Immune Responses

To investigate the humoral immunity, blood samples were collected at 35 and 42 days of age, 7 and 14 days after the first and the second SRBC injections, respectively [38]. Then, the antibody level against SRBC was measured with a hemagglutination assay [37]. The magnitude of the immune response against sheep red blood cells (SRBCs) was determined as described by Pourhossein et al. [39]. Serial dilutions of heat-inactivated serum, at a 2-fold concentration, were prepared in U-bottom microtiter plates using PBS (0.01 mol/L; pH 7.4) for total antibody analysis or PBS containing 1.4% 2-mercaptoethanol for IgG antibody analysis. The titers were expressed based on log2 of the highest dilution showing complete agglutination [40]. The IgM titer was determined by the difference between the total and IgG titers.

To investigate Newcastle disease (NDV) and influenza at 28 and 42 days of age, two birds per pen were selected for blood sampling. Samples were combined, and the serum levels of Newcastle and influenza were evaluated using the hemagglutination inhibition (HI) test according to OIE standards. The experiment utilized 96-well microplates. Initially, 25 microliters of PBS (Pripheral Blood Smear) were added to all wells, followed by 25 microliters of bird serum added to the first well, with subsequent dilutions to the last well. Next, 25 microliters of Newcastle and influenza antigens was added to all wells. The microplate was then shaken mechanically for 1 min and incubated at 25 °C for 30 min. Subsequently, 25 microliters of 1% red blood cells were added to all wells, followed by another 15 s of mechanical shaking and further incubation at 25 °C for 30 min before recording the results. The HI test was performed using a 4-unit antigen (Pasouk, Iran), with titers diluted based on log2. The 1% red blood cells were sourced from SPF (Sun Protection Factor) chicks.

### 2.5. Cecal Microbial Flora

To investigate cecal microbial flora, after slaughtering, the right and left ceca were separated with sterile scissors and the contents were emptied into sterile microtubules. They were held at 20°C until microbial culture was performed to determine the *E. coli* population [41]. Serial dilution (1:10) in distilled water and autoclaving at a pressure of 120 atmospheres were used to dilute the samples [42]. Then, counting of the colonies formed in the most suitable dilution (10-4) was performed to determine the Colony Forming Units (CFU). Due to the large numbers obtained from the bacterial count, to facilitate the calculations, the logarithm base 10 of the numbers was calculated and then used to analyze the data [38].

### 2.6. Statistical Analysis

Statistical analysis was performed in SAS version 9.4 (SAS Institute Inc., Cary, NC, USA). The normal distribution of data was checked by using the UNIVARIATE procedure in SAS, and non-normally distributed data were log-transformed and presented as back-transformed. Data were subjected to ANOVA and analyzed with the GLM procedure in SAS. The statistical model included the experimental diets (CTR, VC200-ASA50, VC200-ASA100, VC400-ASA50 and VC400-ASA100) as a fixed effect. In addition, the normality of the residuals was tested via PROC UNIVARIATE in SAS. The Kenward–Roger statement was used to compute the denominator degrees of freedom. Orthogonal contrasts were used to determine the linear or quadratic effect of experimental diets with VC or AS overall. Data were considered significant at *p* ≤ 0.05, and tendencies were determined at *p* ≤ 0.10 using the PDIFF statement in SAS.

## 3. Results

### 3.1. Feed Intake and Growth Performance

Table 2 presents the impacts of treatment and dosage levels on feed intake, feed conversion ratio, and average daily gain. The treatment exhibited effects on feed intake during the grower period and throughout the entire trial period (*p* = 0.002 and *p* < 0.001, respectively) but with no significant differences between treatments. The control group (CTR) demonstrated lower feed intake levels during both aforementioned phases. The addition of ASA showed a linear effect during the grower (*p* = 0.0001) and overall periods (*p* < 0.0001), along with a quadratic effect (*p* = 0.001). VC supplementation had a linear effect during the grower phase (*p* = 0.0001) and overall period (*p* < 0.001), as well as a quadratic effect during the starter (*p* = 0.009), finisher (*p* = 0.008) and overall periods (*p* < 0.001).

The average daily gain (ADG) was influenced by treatment across the total period and each phase but without differences between them (*p* < 0.0001). In the starter and grower periods, the VC200-ASA50 group showed the highest ADG, while the CTR exhibited the lowest (*p* < 0.0001 and *p* = 0.0003, respectively). In the finisher period, VC200-A100 displayed the highest growth performance and the CTR the lowest (*p* < 0.003). The linear effects of ASA supplementation were observed in all periods, with quadratic effects noted only in the grower and overall periods. Regarding VC, both linear and quadratic effects were recorded throughout all periods with no significant differences between dosages.

In terms of the feed conversion ratio (FCR), a treatment effect was detected during the finisher period (*p* < 0.001) and the total trial period (*p* < 0.0001). The treatment groups had better FCR values, but without differences among them, than the CTR. The addition of ASA displayed linear effects during these periods and quadratic effects throughout the total trial period. VC supplementation exhibited linear effects during the finisher and total periods, along with quadratic effects during the grower and total periods.

The EPI (Table 3) was affected by the treatment, with the CTR having the lowest EPI value (225, *p* < 0.0001). Regarding ASA and VC dose effects, both displayed linear and quadratic effects, with higher values of EPI observed in the ASA100 (316) and VC200 (327) groups.

### 3.2. Serum Parameters and Immunity Parameters

Table 4 presents the analyzed serum parameters, with creatine kinase (CK) being the parameter most influenced by the treatment (*p* = 0.04). Creatine kinase was affected by treatments, with the VC200-ASA100 and VC400-ASA100 groups exhibiting higher levels of CK (35,421.7 and 32,657.25 IU/L, respectively), while the CTR exhibited the lowest value (10,051.00 IU/L). A linear effect was also observed regarding CK, with the ASA100 group displaying the highest value by far, and no differences were noted between the CTR and ASA50 group. The inclusion of ASA demonstrated a linear effect on lactate dehydrogenase (*p* = 0.019), for which an increase in dosage correlated with an increase in the parameter was observed; however, no differences were detected between the CTR and treatment groups.

Table 5 reports serum parameters related to immune response and status. The treatment impacted neutrophil (*p* = 0.025) and lymphocyte concentrations (*p* = 0.032). Neutrophils were lowest in the CTR and VC400-ASA100 groups, and the VC200-ASA50 group exhibited the highest value. For lymphocytes, the VC200-ASA50 group had the lowest value, while CTR and VC400-ASA100 groups had the highest. The intake of ASA at different doses showed a linear effect (*p* = 0.003) on white blood cells, decreasing the levels compared with the CTR, with no differences between the two dosages. Similar effects were observed for VC intake (*p* = 0.005). Additionally, VC intake had a quadratic effect on neutrophils (*p* = 0.003) and lymphocytes (*p* = 0.002). In both cases, the VC400 groups had comparable neutrophil values to the CTR and lower values than the VC200 group, while the opposite was observed for lymphocytes.

Regarding antibody titers, the treatment influenced the influenza antibody titers on day 42 (*p* = 0.001), with the VC400-ASA50 and VC400-ASA100 groups showing the highest values (6.25 and 6.50, respectively) compared with the CTR and other treatments. Antibody titers for sheep red blood cells on day 35 were also affected (*p* = 0.003), with the VC400-ASA100 group having the lowest value (2.75) among all the treatment groups. VC intake had linear effects on influenza antibodies on day 42 and sheep red blood cell antibodies (*p* = 0.001 and 0.039, respectively).

### 3.3. Carcass Traits

Table 6 outlines the carcass traits. The treatment influenced both the live weight of chickens at slaughter and the carcass weight, including both full abdominal carcass weight and empty abdominal carcass weight (*p* < 0.0001). The CTR had the lowest live weights but also eviscerated carcasses (% of body weight), while the VC200-ASA100 treatment group exhibited the highest values for all mentioned parameters. The intake of ASA demonstrated both linear and quadratic effects on the aforementioned parameters, with the ASA100 group showing the highest values for all. Additionally, VC intake affected live weight, carcass weight (full and empty abdominal) and eviscerated carcass as a % of body weight in both linear and quadratic terms, resulting in higher values compared with the untreated group. However, no differences were detected between dosage levels.

In terms of carcass composition, breast and drumstick (in terms of % of body weight, BW) had significantly higher proportions (*p* = 0.0005 and *p* = 0.0004, respectively) in the treatment groups, without differences among them, compared with the CTR. Both ASA and VC exhibited both linear and quadratic effects, resulting in significantly higher values than the zero dosage, and increasing the doses further enhanced these values. Wings were unaffected by treatment, but ASA intake showed a linear effect, decreasing the wing % as ASA dosage increased. The content of abdominal fat, in %BW, was significantly lower in the treatment groups (0.32% BW in VC200-ASA100) compared with the CTR (1.65% BW) (*p* < 0.001). ASA and VC supplements also showed linear and quadratic effects, reducing the relative abdominal fat content with increasing dosage (*p* < 0.0001).

Considering the organs involved in the immune system, the liver (%BW) accounted for equal proportions in the CTR, VC200-ASA100 and VC400-ASA50 groups (2.56, 2.45, 2.49, respectively), with significantly lower values than the highest value of the VC200-ASA100 group (3.27, *p* = 0.018). ASA intake had both linear and quadratic effects, with the higher dose (ASA100) significantly increasing the relative weight of the liver. The relative weight of the thymus (%BW) was lower in the VC400-ASA100 group (0.34% BW) than in the CTR (0.49% BW; *p* < 0.0001). Both ASA and VC intake exhibited linear and quadratic effects, reducing thymus weights. The relative weight of the pancreas was higher in the CTR and VC200-ASA50 group and lower in the VC200-ASA100 group (*p* = 0.005). ASA had a linear effect only at the higher dose, while VC intake showed no differences at different levels. The VC200-ASA100 treatment also led to the lowest value for the bursa of Fabricius (0.05% BW) compared with the highest value in the CTR (0.19, *p* < 0.001). Both ASA and VC supplementation showed linear and quadratic effects.

Considering the relative weights of gastrointestinal tracts (Table 7), the CTR had the highest values for the crop, stomach, gizzard, duodenum, jejunum and rectum (0.44, 0.49, 2.18, 0.72, 1.29, 0.27%BW and *p* = 0.001, 0.001, 0.007, <0.0001, 0.007, <0.0001, respectively). ASA usage decreased all values compared with the CTR. For the crop, duodenum and jejunum, the VC200-ASA50 group’s values were statistically equal to those of the CTR. The VC200-ASA100 group showed the lowest value, along with the VC400-ASA100 group, for the stomach and jejunum. The VC400-ASA50 group had the lowest value for the rectum. VC intake had a linear effect on the crop, stomach, gizzard, duodenum, jejunum, right cecum and rectum, with a quadratic effect on the stomach, gizzard and rectum.

The CTR exhibited the highest cecal *Escherichia coli* count, with all treatment groups significantly differing from both the CTR (8.66 log10 CFU/gr) and each other. Notably, the VC400-ASA100 treatment led to the lowest count (7.55 log10 CFU/gr, *p* < 0.0001). In this instance, both ASA and VC intake demonstrated both linear and quadratic effects, leading to decreased *E. coli* values with higher dosages (*p* < 0.0001).

## 4. Discussion

Dietary supplementation with ASA and VC in broilers significantly enhanced the overall daily growth performance. Although the VC200-A50 dosage yielded the greatest benefits in the initial starter and grower phases, the most favorable performance was observed with the VC400-A50 treatment in the final finishing phase. The results reveal clear positive effects on growth performance with the combined use of ASA and VC, but without noticeable differences between dosages. The improvement in performance can be attributed to both increased feed intake and enhanced feed efficiency, particularly in the final fattening phase, which was characterized by a typically poorer FCR. These findings are consistent with literature studies demonstrating the efficacy of acetylsalicylic acid and vitamin C, either individually or in combination, in reducing weight loss in animals exposed to thermal stress conditions. This is in line with Fathi et al., indicating higher weight gain and an improved FCR can be achieved at an 80 mg ASA dosage [42]. Similar outcomes were corroborated by Al-Obaidi et al. [23] for ASA inclusion at 0.2%. The presumed positive effect of ASA is associated with its anti-inflammatory properties and the mitigation of free radical stress. The absence of performance differences among various dosages corresponds with the findings reported by Stilborn et al. [43] that proved different doses of ASA and VC had immediate beneficial effects in a heat stress condition but variable impacts in terms of growth performance, highlighting the varied effects of different ASA and VC doses. Ahmadu et al. [44] also suggested that the presence of antioxidants, such as vitamin C in this trial, could enhance nutrient digestibility and feed efficiency. Poultry has the ability to synthesize ascorbic acid, but this ability is inadequate under stress conditions, such as high environmental temperatures and high humidity or travel or vaccination [45]. Despite the absence of adverse climate conditions during the trial, the higher dose of VC did not positively affect the chicken growth performance. The 200 mg VC dosage exhibited superior performance, significantly surpassing the control group but not falling below the 400 mg dosage. The excess vitamin C may have been unassimilated and therefore excreted because this vitamin is water-soluble [44]. Kutlu et al. suggested that the supplementation of VC in non-heat-stressed broilers could reduce the broilers’ performances [46]. Also, numerically, there were no differences between treatments, which could lead to a preference for using the lowest dose level to ameliorate the feed costs without reducing the performances.

The utilization of ASA and VC did not affect the cholesterol and triglycerides or VLDL, HDL and LDL serum content, in accordance with the findings of Takahashi et al. and Kutlu et al. for VC [46,47] and Fathi et al. for ASA [42]. However, all the high-dose treatments significantly reduced the abdominal fat compared with the CTR, and this is in line with the findings reported by Omidi et al. [48] when feeding turkeys with VC and ASA.

Considering the well-established correlation between fat deposition in the abdominal area and overall body fatness, the impact of a dietary supplement may be reflected in the total body fat [49]. This assumes significance as a decline in abdominal fat represents an economic loss for chicken meat industry, potentially leading to positive economic implications [27]. The absence of a concurrent reduction in plasma lipids despite the decrease in abdominal fat could be attributed to plasma values within physiological ranges, given the absence of stress conditions such as heat stress [50]. The reduction in oxidative status, supported by acetylsalicylic acid, may have contributed to decreased lipid peroxidation, thereby enhancing growth performance and improving gut conditions that facilitate nutrient absorption. Notably, fat deposition typically increases during the finisher period, aligning with the observed reduction in treatment groups and their improved feed conversion ratio [51].

The treatment did not impact serum lactate dehydrogenase, a cytoplasmic enzyme serving as an oxidative damage indicator responsible for converting pyruvate into lactate [52]. Studies have suggested that aspirin intake under stress conditions could reduce the enzyme serum content outcome of reduced heat stress effects [53,54]. Nonetheless, the ASA intake showed a linear effect that resulted in higher enzyme levels with higher levels of ASA. This finding is in contrast with previous studies and suggests that new studies have to be conducted to investigate a possible negative effect at high dosage levels in non-stress conditions. Lactate dehydrogenase is also an index of liver damage, and the trial results showed an increase in the relative weight of the liver at higher ASA levels (100 mg). No effect was detected from the intake of VC, as reported by Fahmideh et al. [55]. Creatine kinase (CK) is an indicator of muscle damage as well as liver damage and is used to predict wooden breast in poultry [56]. It showed an increase in groups treated with the higher ASA level (100 mg) compared with the untreated group and the group treated with the lower ASA level (50 mg). While ASA is generally considered safe in poultry nutrition, information on tolerance to high levels of acetylsalicylic acid (400 mg) suggests that it leads to an increase in the liver-to-body weight ratio, which is consistent with the presented study [55]. Mohan et al. [57] showed that at dose of 10 mg/kg, ASA could be toxic for broilers. Given the lack of alteration in liver enzymes, further investigation must be undertaken.

At the carcass level, the increase in the relative weight of breasts and drumsticks and the decrease in that of heads could be related to a decrease in abdominal fat, resulting in an improvement in the commercial carcass cut most preferred by consumers. Upon examining the gastrointestinal tract, the treated groups presented a decreased relative weight compared with the control group. This finding supports the higher contributions of the breast and drumstick and that a higher FCR represents a better health status. Also, in this case, the best performances were achieved with a low dose of ASA and a higher dose of VC. Due to the antioxidant and radical scavenging properties of aspirin [58], it could protect the endothelium from oxidative stress, and its combination with vitamin C could enhance the attenuating oxidative stress function. Rajani et al. observed a reduction in the left cecum compared with the control in the ASA group [59]. The integration of vitamin C also had a decreasing effect on the gastrointestinal system’s relative weight, and the highlighted positive effect on gut morphology and intestinal mucosa could have supported the better growth performances [17,60].

Intestinal microorganisms are pivotal in shaping the well-being and intestinal conditions of broiler chickens. At the intestinal level, in all the treated groups, the *E. coli* count was lower, significantly decreasing with increasing levels of both ASA and VC. This aligns with the findings of Nosrati et al. and Fahmideh et al. when using vitamin C [55,61] and those of Konieczka et al., which indicated the potential of ASA and vitamin E in modulating the inflammatory cascades in LPS inflammation [62].

The higher dose of vitamin C led to an increase in the count of influenza antibodies on day 42 without ASA level effects. Conversely, the ASA400-A100 group showed the lowest count of sheep red blood cells, indicating that a higher level of vitamin C decreases the titer, supported by the linear effect of VC intake. This is in line with what has been reported by several authors who used ascorbic acid to test its effect on antibody titers (i.e., SBRC, IBD and Newcastle disease) [62,63,64]. This may be attributed to the vitamin’s influence on the immune system through the modulation of cell-mediated and antibody-mediated responses, immunoregulation, anti-inflammatory effects and enhancing responses to vaccines [65,66,67,68]. However, as determined by Shojadoost et al., there are inconsistent results regarding the effect of VC on immune responses, which could be due to several factors (i.e., dose, environmental conditions, vaccination schedule), so the dietary intake may be beneficial, but the mechanisms of antioxidant effects must be investigated [66]. The trial did not show ASA’s effect on titer count, but the recognized immunoregulatory potential of aspirin in relation to immune tolerance (e.g., tolerogenic activity in dendritic cells, determining the response of naive T cells to regulatory phenotypes), and traits modulating innate and adaptive immune responses have been reported [69]. White blood cells were not affected by the treatments in this trial, but ASA and VC both showed a linear effect, reducing the WBC in treated groups. The percentage of neutrophils decreased with increasing levels of VC, and thus the VC200-ASA group had clearly higher values; however, the VC400-ASA100 group had equal values to the control group. Neutrophils are key players in innate immunity, and after their extravasation from the circulatory system toward the site of infection or tissue damage, they produce cytokines. ASA is reported to suppress the neutrophil-mediated innate immune responses by decreasing their extravasation or decreasing the adherence of neutrophils to the endothelial lining [69]. The non-clear results suggest the need for further investigations. A similar trend was observed in lymphocyte concentrations. Lymphocytes play a pivotal role in the regulation of adaptive immune responses against different protein antigens. The proposed results indicated a decreasing effect on lymphocytes with the intake of ascorbic acid at a lower dose, warranting further investigation, as this does not align with the hypothesis that ascorbic acid supports immune systems by modulating the activities of phagocytes, the production of lymphocytes and cytokines and the number of cell adhesion molecules in monocytes [68]. The organs involved in the immune system, particularly the thymus, pancreas and bursa of Fabricius, were affected by the treatment, and their relative weights were decreased in comparison with those of the control group. The dose of ASA and VC had no effect on the reduction, but the VC200-ASA100 group had the lowest pancreas and bursa relative weights, and the VC400-ASA100 group had the lowest thymus one. These same groups also had higher liver relative weights and concentrations of lymphocytes and neutrophils compared with the control group. These findings might be explained by a reduction in organ weight due to an increase in other carcass parts, shedding light on the absence of an increase in serum immune system cells. However, these observations do not align with references that predominantly report a positive effect of ASA intake on the immune response, improving the ratios of the thymus, spleen and bursa to body weight, which could be atrophied in stress conditions [21,33,70,71]. The unclear effect of treatment could also be attributed to the absence of a particular stress during the trial.

Table 8 reports the main trial findings considering the effect of the combination of ASA and VC and the dose effect of the two.

## 5. Conclusions

Improving production performance in broiler farming and enhancing the quality of the final product are crucial aspects for both consumers and scientific research.

The ASA-VC treatment improved growth performance and the EPI. A high dosage of ASA (100), despite yielding the best results in live weight and *E. coli* reduction, demonstrated an increase in lactate dehydrogenase and creatine kinase levels. The two dosages of VC showed no differences, suggesting the effectiveness of a low dosage under non-stress conditions. Thus, the efficacy of these two compounds was observed even under normal rearing conditions, but the results underscore the need for a more thorough investigation into the minimum effective dosage for both substances and an exploration of the potential variations under specific stress conditions.

## Figures and Tables

**Table 1:** Ingredients, chemical composition and energy of the diets used during the starter period (1 to 10 d of age), grower period (11 to 24 d of age) and finisher period (25 to 42 d of age).

Ingredients (g/kg as Fed)	Starter Diet	Grower Diet	Finisher Diet
	1st–10th Days	11st–24th Days	25th–42nd Days
Corn	47.03	59.60	65.99
Wheat	5.58	5.00	5.00
Soybean meal (44% crude protein)	29.02	16.15	10.28
Corn gluten	10.00	11.48	11.50
Soybean oil	3.50	3.40	3.09
Limestone	1.45	1.23	1.00
Di-calcium phosphate	1.95	1.80	1.83
Salt	0.20	0.20	0.20
Vitamin and mineral supplements ^1^	0.50	0.50	0.50
DL-methionine	0.52	0.58	0.57
L-lysine hydrochloride	0.25	0.06	0.04
Metabolizable energy (kcal/kg)	2950	3000	3050
Crude protein (%)	22	20	19
Lysine (%)	1.3	1.2	1.1
Metionine (%)	0.56	0.54	0.52
Met + Cys (%)	0.92	0.90	0.88
Calcium (%)	1	0.95	0.92
Available phosphorus	0.52	0.47	0.41

^1^ The amount of vitamins and minerals per kg of the final diet: vitamin A, 9000 IU; vitamin D3, 3000 IU; vitamin E, 18 IU; vitamin K3, 3 mg; vitamin B1 (Thiamine), 1/8 mg; vitamin B2 (Riboflavin), 6 mg; vitamin B6 (Pyridoxine), 3 mg; vitamin B12 (Cyanocobalamin), 0/012 mg; vitamin B3 (Niacin), 30 mg; vitamin B9 (Folic acid), 1 mg; vitamin H3 (Biotin), 0/24 mg; vitamin B5 (Pantothenic acid), 10 mg; Choline, 100 mg; Mn, 100 mg; Zinc, 80 mg; Iron, 10 mg; Cu, 1 mg; I, 2 mg.

**Table 2:** Effect of treatment and dose of treatment on feed intake, feed conversion ratio (FCR) and average daily gain (ADG) during starter period (1–10 days), grower period (11–24 days), finisher period (25–42 days) and total period of the trial.

Items		Feed Intake Starter Period	Feed Intake Grower Period	Feed Intake Finisher Period	Feed Intake Total Period	FCR Starter Period	FCR Grower Period	FCR Finisher Period	FCR Total Period	ADG Starter Period	ADG Grower Period	ADG Finisher Period	ADG total Period
		g/Head/Day	g/Head/Day	g/Head/Day	g/Head/Day	g Feed/g Weight	g Feed/g Weight	g Feed/g Weight	g Feed/g Weight	g/Head/Day	g/Head/Day	g/Head/Day	g/Head/Day
CTR		18.10	48.40 ^a^	144.83	82.51 ^a^	1.88	1.48	2.12 ^a^	1.94 ^a^	9.65 ^a^	32.71 ^a^	68.57 ^a^	42.59 ^a^
VC200-ASA50		21.95	66.86 ^b^	162.92	97.33 ^b^	1.55	1.23	2.07 ^a^	1.76 ^b^	14.20 ^b^	54.32 ^b^	78.70 ^b^	55.22 ^b^
VC200-ASA100		20.70	68.36 ^b^	165.17	98.50 ^b^	1.70	1.39	1.88 ^b^	1.73 ^b^	12.20 ^c^	49.36 ^c^	87.94 ^c^	57.05 ^b^
VC400-ASA50		18.85	68.03 ^b^	158.65	95.16 ^b^	1.81	1.45	1.90 ^b^	1.76 ^b^	10.80 ^ac^	47.00 ^c^	83.47 ^bc^	54.01 ^b^
VC400-ASA100		20.00	77.25 ^b^	154.26	96.62 ^b^	1.48	1.57	1.86 ^b^	1.76 ^b^	13.50 ^bc^	49.00 ^c^	83.00 ^bc^	55.12 ^b^
*p*-value		0.078	0.002	0.081	<0.0001	0.166	0.075	0.001	<0.0001	0.0003	<0.0001	0.003	<0.0001
SEM		0.94	3.97	5.03	1.61	0.12	0.08	0.04	0.02	0.50	1.15	2.82	1.09
ASA0		18.10	48.40 ^a^	144.83 ^a^	82.51 ^a^	1.88	1.48	2.12 ^a^	1.94 ^a^	9.65 ^a^	32.71 ^a^	68.57 ^a^	42.59 ^a^
ASA50		20.40	67.44 ^b^	160.78 ^b^	96.25 ^b^	1.68	1.34	1.99 ^b^	1.76 ^b^	12.50 ^b^	50.66 ^b^	81.09 ^b^	54.62 ^b^
ASA100		20.35	72.80 ^b^	159.72 ^b^	97.56 ^b^	1.59	1.48	1.86 ^c^	1.74 ^b^	12.85 ^b^	49.18 ^b^	85.47 ^b^	56.08 ^b^
SEM		0.73	2.85	3.63	1.13	0.09	0.06	0.03	0.02	0.59	1.18	2.05	0.78
*p*-value	L	0.094	0.0001	0.030	<0.0001	0.087	0.990	0.001	<0.0001	0.006	<0.0001	0.0002	<0.0001
	Q	0.024	0.087	0.094	0.001	0.661	0.103	0.907	0.002	0.125	<0.0001	0.153	<0.0001
VC0		18.10 ^a^	48.40 ^a^	144.83 ^a^	82.51 ^a^	1.88	1.48	2.12 ^a^	1.94 ^a^	9.65 ^a^	32.71 ^a^	68.57 ^a^	42.59 ^a^
VC200		21.33 ^bc^	67.61 ^b^	164.04 ^b^	97.92 ^b^	1.62	1.31	1.98 ^b^	1.75 ^b^	13.20 ^b^	51.84 ^b^	83.32 ^b^	56.13 ^b^
VC400		19.43 ^ac^	72.64 ^b^	156.46 ^a^	95.89 ^b^	1.65	1.51	1.88 ^b^	1.79 ^b^	12.15 ^b^	48.00 ^c^	83.24 ^b^	54.57 ^b^
SEM		0.66	2.87	3.39	1.10	0.09	0.06	0.04	0.02	0.56	1.00	2.19	0.77
*p*-value	L	0.260	0.0001	0.064	<0.001	0.169	0.767	0.001	<0.001	0.020	<0.001	0.001	<0.001
	Q	0.009	0.079	0.008	<0.001	0.272	0.026	0.673	0.0002	0.006	<0.001	0.020	<0.001

CFR: feed conversion ratio; ADG: average daily gain; CTR: control group; VC: vitamin C; ASA: acetylsalicylic acid; L: linear effect; Q: quadratic effect. Means within each column with different superscripts are statistically different (*p* ≤ 0.05).

**Table 3:** Effect of treatment and dose of treatment on European Production Index (EPI) during total period of the trial.

Items		European Production Index
CTR		224 ^a^
VC200-ASA50		319 ^b^
VC200-ASAS100		336 ^b^
VC400-ASA50		312 ^b^
VC400-ASA100		320 ^b^
SEM		9.00
*p*-value		<0.0001
ASA0		225 ^a^
ASA50		315 ^b^
ASA100		328 ^b^
SEM		6.33
*p*-value	L	<0.0001
	Q	0.0002
VC0		2245 ^a^
VC200		327 ^b^
VC400		316 ^b^
SEM		6.41
*p*-value	L	<0.001
	Q	<0.001

CTR: control group; VC: vitamin C; ASA: acetylsalicylic acid; L: linear effect; Q: quadratic effect. Means within each column with different superscripts are statistically different (*p* ≤ 0.05).

**Table 4:** Effect of treatment and dose of treatment on serum parameters.

Items		Glucose	Cholesterol	Triglycerides	VLDL	HDL	LDL	Alkaline Phosphatase	Alanine Transferase	Lactate Dehydrogenase	Creatine Kinase	Total Protein	Albumin	Globulin
		mg/dL	mg/dL	mg/dL	mg/dL	mg/dL	mg/dL	U/L	IU/L	IU/L	IU/L	g/dL	g/dL	g/dL
CTR		197.75	131.25	76.25	15.28	72.25	36.00	5048.25	239.25	4280.00	10,051.00 ^a^	3.80	2.48	1.33
VC200-ASA50		165.25	163.50	55.75	11.15	90.25	50.00	3953.25	404.25	5008.50	20,438.25 ^a^	3.80	1.88	1.93
VC200-ASA100		190.75	162.75	93.75	18.75	87.00	43.75	3906.00	509.25	6357.75	35,421.75 ^ab^	3.88	2.03	1.85
VC400-ASA50		176.50	148.75	85.00	17.00	84.00	41.50	4662.00	325.75	4798.50	21,383.25 ^a^	3.48	1.88	1.60
VC400-ASA100		153.50	142.25	67.25	13.45	78.50	40.25	3897.50	436.50	6142.50	32,657.25 ^ab^	3.83	2.15	1.68
*p*-value		0.492	0.201	0.791	0.420	0.321	0.414	0.360	0.156	0.174	0.040	0.934	0.725	0.246
SEM		19.15	10.53	22.89	4.58	6.30	5.02	888.81	74.58	660.03	5647.41	0.36	0.35	0.19
ASA0		197.75	131.25	76.25	15.28	72.25	36.00	5048.25	239.25	4280.00 ^a^	10,051.00 ^a^	3.80	2.48	1.33
ASA50		170.88	156.13	70.25	14.08	87.13	45.75	4307.63	365.00	4903.50 ^a^	20,910.75 ^a^	3.64	1.88	1.76
ASA100		172.13	152.50	80.50	16.10	82.75	42.00	3901.75	472.88	6250.13 ^b^	34,039.50 ^b^	3.85	2.09	1.76
SEM		13.57	7.63	15.94	3.19	4.38	3.51	596.58	51.21	439.91	3767.77	0.24	0.23	0.13
*p*-value	L	0.291	0.127	0.880	0.883	0.184	0.338	0.283	0.017	0.019	0.002	0.906	0.346	0.076
	Q	0.444	0.176	0.709	0.077	0.115	0.165	0.835	0.897	0.543	0.823	0.562	0.201	0.232
VC0		197.75	131.25	76.25	15.28	72.25	36.00	5048.25	239.25	4280.00	10,051.00	3.80	2.48	1.33
VC200		178.00	163.25	74.75	14.95	88.63	46.88	3929.63	456.75	5683.13	27,930.00	3.84	1.95	1.89
VC400		165.00	145.50	76.13	15.23	81.25	40.88	4279.75	381.13	5470.50	27,020.25	3.65	2.01	1.64
SEM		13.38	7.04	16.03	3.21	4.25	3.42	597.62	52.88	495.51	4386.49	0.24	0.23	0.13
*p*-value	L	0.176	0.259	0.997	0.993	0.239	0.422	0.468	0.140	0.183	0.039	0.723	0.268	0.172
	Q	0.851	0.017	0.947	0.944	0.050	0.080	0.366	0.051	0.235	0.124	0.728	0.354	0.027

VLDL: very-low-density lipoprotein; HDL: high-density lipoprotein; LDL: low-density lipoprotein; CTR: control group; VC: vitamin C; ASA: acetylsalicylic acid; L: linear effect; Q: quadratic effect. Means within each column with different superscripts are statistically different (*p* ≤ 0.05).

**Table 5:** Effect of treatment and dose of treatment on immunity parameters.

Items		White Blood Cells	Neutrophils	Lymphocytes	Eosinophils	Antibodies against Newcastle (lg2) (28 d)	Antibodies against Newcastle (lg2) (42 d)	Antibodies against Influenza (lg2) (28 d)	Antibodies against Influenza (lg2) (42 d)	Antibodies against Sheep Red Blood Cells (35 d)	Antibodies against Sheep Red Blood Cells (42 d)
		(*n* × 10^3^/mL)	%	%	%						
CTR		5250	14.25 ^a^	80.00 ^a^	5.50	4.00	6.00	2.50	4.50 ^a^	5.50 ^a^	6.75
VC200-ASA50		2175	43.50 ^b^	54.00 ^b^	2.50	2.25	4.50	2.75	4.25 ^a^	5.25 ^a^	6.75
VC200-ASA100		1450	29.75 ^ab^	63.25 ^ab^	7.00	2.50	4.00	2.50	4.50 ^a^	5.75 ^a^	7.50
VC400-ASA50		1750	19.00 ^ab^	76.25 ^ab^	4.75	3.25	5.50	2.75	6.25 ^b^	5.00 ^a^	7.50
VC400-ASA100		1250	16.50 ^a^	78.75 ^a^	4.50	3.50	5.50	2.75	6.50 ^b^	2.75 ^b^	6.50
*p*-value		0.064	0.025	0.032	0.448	0.306	0.098	0.896	0.001	0.003	0.143
SEM		982.75	6.20	6.04	1.65	0.63	0.53	0.27	0.38	0.47	0.33
ASA0		5250 ^a^	14.25	80.00	5.50	4.00	6.00	2.50	4.50	5.50	6.75
ASA50		1962 ^b^	31.25	65.13	3.63	2.75	5.00	2.75	5.25	5.13	7.13
ASA100		1350 ^b^	23.13	71.00	5.75	3.00	4.75	2.63	5.50	4.25	7.00
SEM		655.23	5.33	5.19	1.17	0.45	0.42	0.18	0.42	0.48	0.27
*p*-value	L	0.003	0.350	0.331	0.903	0.216	0.101	0.692	0.192	0.151	0.595
	Q	0.141	0.093	0.149	0.214	0.224	0.505	0.440	0.662	0.699	0.487
VC0		5250 ^a^	14.25 ^a^	80.00 ^a^	5.50	4.00 ^a^	6.00 ^a^	2.50	4.50 ^a^	5.50 ^a^	6.75
VC200		1812 ^b^	36.63 ^b^	58.63 ^b^	4.75	2.38 ^b^	4.25 ^b^	2.63	4.38 ^a^	5.50 ^a^	7.13
VC400		1500 ^b^	17.75 ^a^	77.50 ^a^	4.63	3.38 ^a^	5.50 ^a^	2.75	6.38 ^b^	3.88 ^b^	7.00
SEM		661.43	4.45	4.18	1.23	0.42	0.36	0.18	0.25	0.42	0.27
*p*-value	L	0.005	0.656	0.734	0.685	0.400	0.432	0.423	0.001	0.039	0.595
	Q	0.092	0.003	0.002	0.850	0.030	0.006	1.000	0.006	0.161	0.487

CTR: control group; VC: vitamin C; ASA: acetylsalicylic acid; linear effect; Q: quadratic effect. Means within each column with different superscripts are statistically different (*p* ≤ 0.05).

**Table 6:** Effect of treatment and dose of treatment on carcass traits and organs.

Items		LW	FACW	EACW	EC	Breast	Drumsticks	Wings	Head	Liver	Thymus	Pancreas	Bursa of Fabricius	Spleen	Abdominal Fat
		g	g	g	% BW	% BW	% BW	% BW	% BW	% BW	% BW	% BW	% BW	% BW	% BW
CTR		2267 ^a^	1869 ^a^	1596 ^a^	0.79 ^a^	26.93 ^a^	22.00 ^a^	8.27	2.29 ^a^	2.56 ^a^	0.49 ^a^	0.31 ^a^	0.19 ^a^	0.13	1.65 ^a^
VC200-ASA50		2717 ^b^	2339 ^b^	2061 ^b^	0.82 ^b^	31.80 ^b^	27.03 ^b^	7.32	2.03 ^b^	2.45 ^a^	0.37 ^b^	0.29 ^a^	0.08 ^b^	0.11	0.44 ^b^
VC200-ASA100		3062 ^c^	2664 ^c^	2370 ^c^	0.83 ^b^	33.02 ^b^	28.59 ^b^	7.17	1.83 ^c^	3.27 ^b^	0.35 ^b^	0.21 ^b^	0.05 ^c^	0.10	0.32 ^b^
VC400-ASA50		2872 ^bc^	25,034 ^bc^	2231 ^bc^	0.83 ^b^	31.69 ^b^	28.35 ^b^	7.76	1.98 ^bc^	2.49 ^a^	0.38 ^b^	0.27 ^ac^	0.07 ^bc^	0.11	0.33 ^b^
VC400-ASA100		2892 ^bc^	2533 ^bc^	2255 ^bc^	0.84 ^b^	32.44 ^b^	27.89 ^b^	7.26	1.97 ^bc^	2.96 ^ab^	0.34 ^c^	0.23 ^bc^	0.07 ^bc^	0.10	0.33 ^b^
*p*-value		<0.0001	<0.0001	<0.0001	<0.0001	0.0005	0.0004	0.161	0.003	0.018	<0.0001	0.005	<0.0001	0.369	<0.0001
SEM		68.26	67.29	65.97	0.01	0.78	0.86	0.33	0.07	0.17	0.01	0.02	0.01	0.01	0.05
ASA0		2267 ^a^	1869 ^a^	1596 ^a^	0.79 ^a^	26.93 ^a^	22.00 ^a^	8.27 ^a^	2.29 ^a^	2.56 ^a^	0.49 ^a^	0.31 ^a^	0.19 ^a^	0.13	1.65 ^a^
ASA50		2795 ^b^	2425 ^b^	2146 ^b^	0.83 ^b^	31.75 ^b^	27.69 ^b^	7.54 ^ac^	2.00 ^b^	2.47 ^a^	0.37 ^b^	0.28 ^a^	0.07 ^b^	0.11	0.39 ^b^
ASA100		2977 ^c^	2598 ^c^	2313 ^c^	0.83 ^b^	32.73 ^b^	28.24 ^b^	7.22 ^bc^	1.90 ^b^	3.11 ^b^	0.35 ^b^	0.22 ^b^	0.06 ^b^	0.10	0.33 ^b^
SEM		2865.69	51.47	50.36	0.00	0.53	0.60	0.23	0.05	0.12	0.01	0.01	0.00	0.01	0.03
*p*-value	L	<0.0001	<0.0001	<0.0001	<0.0001	<0.0001	<0.0001	0.016	0.000	0.018	<0.0001	0.0003	<0.0001	0.037	<0.0001
	Q	0.025	0.013	0.011	0.002	0.014	0.005	0.503	0.165	0.036	0.003	0.529	<0.0001	0.722	<0.0001
VC0		2267 ^a^	1869 ^a^	1596 ^a^	0.79 ^a^	26.93 ^a^	22.00 ^a^	8.27	2.29 ^a^	2.56	0.49 ^a^	0.31 ^a^	0.19 ^a^	0.13	1.65 ^a^
VC200		2890 ^b^	2502 ^b^	2215 ^b^	0.83 ^b^	32.41 ^b^	27.81 ^b^	7.24	1.93 ^b^	2.86	0.36 ^b^	0.25 ^b^	0.07 ^b^	0.10	0.38 ^b^
VC400		2882 ^b^	2518 ^b^	2243 ^b^	0.84 ^b^	32.06 ^b^	28.12 ^b^	7.51	1.98 ^b^	2.73	0.36 ^b^	0.25 ^b^	0.07 ^b^	0.10	0.33 ^b^
SEM		61.74	59.67	57.72	0.00	0.55	0.61	0.23	0.05	0.16	0.01	0.02	0.01	0.01	0.03
*p*-value	L	<0.001	<0.001	<0.001	<0.001	<0.001	<0.001	0.073	0.002	0.562	<0.001	0.027	<0.001	0.091	<0.001
	Q	0.001	0.001	0.001	0.004	0.001	0.003	0.048	0.007	0.332	0.0004	0.140	<0.001	0.268	<0.001

CTR: control group; VC: vitamin C; ASA: acetylsalicylic acid; LW: live weight; FACW: full abdominal carcass weight; EACW: empty abdominal carcass weight; EC: eviscerate carcass; BW: body weight; L: linear effect; Q: quadratic effect. Means within each column with different superscripts are statistically different (*p* ≤ 0.05).

**Table 7:** Effect of treatment and dose of treatment on gastrointestinal tracts traits and cecal *E. coli* count.

Items		Crop	Stomach	Gizzard	Duodenum	Jejunum	Ileum	Colon	Right Cecum	Left Cecum	Rectum	*E. coli*
		% BW	% BW	% BW	% BW	% BW	% BW	% BW	% BW	% BW	% BW	log10 CFU/gr
CTR		0.44 ^a^	0.49 ^a^	2.18 ^a^	0.72 ^a^	1.29 ^a^	0.54	0.38	0.19	0.19	0.24 ^ac^	8.66 ^a^
VC200-ASA50		0.43 ^a^	0.42 ^b^	1.65 ^b^	0.69 ^a^	1.21 ^ac^	0.57	0.37	0.19	0.19	0.22 ^b^	7.92 ^b^
VC200-ASA100		0.37 ^b^	0.35 ^c^	1.64 ^b^	0.58 ^b^	0.99 ^b^	0.53	0.29	0.15	0.15	0.18 ^ab^	7.84 ^c^
VC400-ASA50		0.30 ^cd^	0.37 ^cd^	1.80 ^b^	0.52 ^b^	1.13 ^bc^	0.45	0.31	0.15	0.15	0.17 ^c^	7.86 ^d^
VC400-ASA100		0.35 ^bc^	0.36 ^c^	1.77 ^b^	0.59 ^b^	1.11 ^bc^	0.50	0.32	0.16	0.16	0.18 ^ac^	7.55 ^e^
*p*-value		0.001	0.001	0.007	<0.0001	0.007	0.200	0.084	0.054	0.091	<0.0001	<0.0001
SEM		0.02	0.02	0.10	0.02	0.05	0.03	0.02	0.01	0.01	0.15	0.00
ASA0		0.44	0.49 ^a^	2.18 ^a^	0.72 ^a^	1.29 ^a^	0.54	0.38	0.19	0.19	0.24 ^a^	8.66 ^a^
ASA50		0.37	0.39 ^b^	1.72 ^b^	0.61 ^b^	1.17 ^b^	0.51	0.34	0.17	0.17	0.19 ^b^	7.89 ^b^
ASA100		0.36	0.36 ^b^	1.71 ^b^	0.58 ^b^	1.05 ^c^	0.51	0.31	0.15	0.16	0.18 ^b^	0.70 ^c^
SEM		0.02	0.01	0.07	0.02	0.04	0.03	0.02	0.01	0.01	0.01	0.04
*p*-value	L	0.041	<0.0001	0.001	0.004	0.002	0.595	0.032	0.028	0.053	0.000	<0.0001
	Q	0.254	0.188	0.024	0.191	0.964	0.613	0.821	0.957	0.871	0.126	<0.0001
VC0		0.44 ^a^	0.49 ^a^	2.18 ^a^	0.72 ^a^	1.29 ^a^	0.54	0.38	0.19	0.19	0.24 ^a^	8.66 ^a^
VC200		0.40 ^a^	0.39 ^b^	1.64 ^b^	0.63 ^b^	1.10 ^b^	0.55	0.33	0.17	0.17	0.20 ^b^	7.88 ^b^
VC400		0.33 ^b^	0.36 ^b^	1.79 ^b^	0.56 ^c^	1.12 ^b^	0.48	0.31	0.16	1.58	0.17 ^c^	7.705 ^c^
SEM		0.02	0.02	0.06	0.02	0.04	0.02	0.07	0.01	0.01	0.01	0.04
*p*-value	L	0.001	0.000	0.002	0.000	0.029	0.135	0.059	0.050	0.082	<0.001	<0.001
	Q	0.362	0.077	0.001	0.811	0.087	0.211	0.494	0.735	0.613	0.035	<0.001

CTR: control group; VC: vitamin C; ASA: acetylsalicylic acid; BW: body weight; L: linear effect; Q: quadratic effect. Means within each column with different superscripts are statistically different (*p* ≤ 0.05).

**Table 8:** The effect of the ASA-VC treatment and the impact of higher dosages of individual substances. The effects are identified as both positive (↑) and negative (↓) compared with the untreated control group.

Treatment	Feed Intake	FCR	ADG	EPI	Lactate Dehydrogenase	Creatine Kinase	Antibody Titers SRBC (35 d)	LW	Eviscerated Carcass	Abdominal Fat	*E. coli*
VC-ASA	↑	↑	↑	↑		↓		↑	↑	↑	↑
ASA100					↓	↓		↑			↑
VC400							↓				↑

CTR: control group; VC: vitamin C; ASA: acetylsalicylic acid; FCR: feed conversion ratio, ADG: average daily gain, EPI: European Production Index; SRBC: sheep blood red cell LW: live body weight.

## Data Availability

Data are contained within the article.

## References

[1] Hosseini-Asl S.S., Pourfarzi F., Barzegar A., Mazani M., Farahmand N., Niasti E., Yazdanbod A., Didevar R., Akhavan H., Malekzadeh R. (2013). Decrease in gastric cancer susceptibility by MTHFR C677T polymorphism in Ardabil Province, Iran. Turk. J. Gastroenterol..

[2] Higgins S., Higgins J., Wolfenden A., Henderson S., Torres-Rodriguez A., Tellez G., Hargis B. (2008). Evaluation of a lactobacillus-based probiotic culture for the reduction of salmonella enteritidis in neonatal broiler chicks. Poult. Sci..

[3] Das L., Bhaumik E., Raychaudhuri U., Chakraborty R. (2012). Role of nutraceuticals in human health. J. Food Sci. Technol..

[4] Lennernäs M., Fjellström C., Becker W., Giachetti I., Schmitt A., De Winter A.M., Kearney M. (1997). Influences on food choice perceived to be important by nationally-representative samples of adults in the European Union. Eur. J. Clin. Nutr..

[5] Rokade J., Bhanja S., Shinde A., Sajjad D., Bhaisare B., Mandal A.B. (2017). Evaluation of aspirin (ASA) in broiler chicken during hot dry summer using zoo technical, molecular and physio-biochemical tools. Indian J. Anim. Res..

[6] Kucuk O., Sahin N., Sahin K., Gursu M.F., Gulcu F., Ozcelik M., Issi M. (2003). Egg production, egg quality, and lipid peroxidation status in laying hens maintained at a low ambient temperature (6 °C) and fed a vitamin C and vitamin E-supplemented diet. Vet. Med..

[7] T. Baltazar M., J. Dinis-Oliveira R., A. Duarte J., L. Bastos M., Carvalho F. (2011). Antioxidant Properties and Associated Mechanisms of Salicylates. Curr Med Chem..

[8] Hajati H., Hassanabadi A., Golian A., Nassiri-Moghaddam H., Nassiri M.R. (2015). The effect of grape seed extract and vitamin C feed supplementation on some blood parameters and HSP70 gene expression of broiler chickens suffering from chronic heat stress. Ital. J. Anim. Sci..

[9] Cinar M., Yildirim E., Yigit A., Yalcinkaya I., Duru O., Kisa U., Atmaca N. (2014). Effects of dietary supplementation with vitamin C and vitamin E and their combination on growth performance, some biochemical parameters, and oxidative stress induced by copper toxicity in broilers. Biol. Trace Element Res..

[10] Dabrowski K., Ciereszko A. (2001). Ascorbic acid and reproduction in fish: Endocrine regulation and gamete quality. Aquac. Res..

[11] Gan L., Fan H., Nie W., Guo Y. (2018). Ascorbic acid synthesis and transportation capacity in old laying hens and the effects of dietary supplementation with ascorbic acid. J. Anim. Sci. Biotechnol..

[12] Gursu M., Onderci M., Gulcu F., Sahin K. (2004). Effects of vitamin C and folic acid supplementation on serum paraoxonase activity and metabolites induced by heat stress in vivo. Nutr. Res..

[13] Mirzapor S.S., Salari S., Mirzadeh K., Aghaei A. (2016). Effect of different levels of vitamin C and L-carnitine on performance and some blood and immune parameters of broilers under heat stress. Iran. J. Anim. Sci. Res..

[14] Attia Y.A., Al-Harthi M.A., El-Shafey A.S., Rehab Y.A., Kim W.K. (2017). Enhancing tolerance of broiler chickens to heat stress by supplementation with vitamin E, vitamin C and/or probiotics. Ann. Anim. Sci..

[15] Jahejo A.R., Rajput N., Tian W.-X., Naeem M., Kalhoro D.H., Kaka A., Niu S., Jia F.-J. (2019). Immunomodulatory and growth promoting effects of basil (*Ocimum basilicum*) and ascorbic acid in heat stressed broiler chickens. Pak. J. Zool..

[16] Angani M. (2017). Ascorbic acid supplementation effect on haematology and oxidative stress parameters of broiler chicken during the hot-dry season in southern guinea savannah. J. Poult. Res..

[17] Nosrati M., Javandel F., Camacho L.M., Khusro A., Cipriano M., Seidavi A., Salem A.Z.M. (2017). The effects of antibiotic, probiotic, organic acid, vitamin C, and Echinacea purpurea extract on performance, carcass characteristics, blood chemistry, microbiota, and immunity of broiler chickens. J. Appl. Poult. Res..

[18] Griffin H.D., Windsor D., Whitehead C.C. (1991). Changes in lipoprotein metabolism and body composition in chickens in response to divergent selection for plasma very low density lipoprotein concentration. Br. Poult. Sci..

[19] Zaghari M., Mohiti Asli M. (2011). Effects of dietary vitamin E or C on layer hen performance, serum parameters and egg cholesterol content. J. Vet. Res..

[20] Hilário M.O.E., Terreri M.T., Len C.A. (2006). Antiinflamatórios não-hormonais: Inibidores da ciclooxigenase 2. J. Pediatr..

[21] Alagawany M., Farag M., El-Hack M.A., Dhama K., Fowler J. (2017). Use of acetylsalicylic acid as a feed additive in poultry nutrition. World’s Poult. Sci. J..

[22] Ghalib R., Falah A., Ahmad A. (2011). Ulcerogenic effects of NSAIDs on gastric mucosa: Comparison between selective and non-selective Cox2 inhibitors; indomethacin VS rofecoxib. Med. J. Babylon.

[23] Al-Obaidi A.F., Al-Shadeedi M.S. (2010). Effect of dietary aspirin for reducing ascites and enhancing productive performance of broilers reared in high density. AL-Qadisiyah J. Vet. Med. Sci..

[24] Glick B. (1963). Influence of acerylslicyclis acid on the body-temperature of heat stressed chickens. Nature.

[25] Nakaue H.S., Weber C.W., Reid B.L. (1967). The influence of acetyl-salicylic acid on growth and some respiratory enzymes in broiler chicks. Exp. Biol. Med..

[26] El-Kholy M.S., El-Hindawy M.M., Alagawany M., El-Hack M.E.A., El-Sayed S.A. (2018). Use of acetylsalicylic acid as an allostatic modulator in the diets of growing Japanese quails exposed to heat stress. J. Therm. Biol..

[27] Rohleder N. (2008). Preventing acute stress-induced inflammatory disinhibition by aspirin: What does it tell us about the mechanism?. Brain Behav. Immun..

[28] Rubio-García M.E., Rubio-Lozano M.S., Ponce-Alquicira E., Rosario-Cortes C., Nava G.M., Castañeda-Serrano M.P. (2015). Improving appearance and microbiologic quality of broiler carcasses with an allostatic modulator. Poult. Sci..

[29] Valko M., Morris H., Cronin M.T.D. (2005). Metals, toxicity and oxidative stress. Curr. Med. Chem..

[30] Tras B., Inal F., Bas A., Altunok V., Elmas M., Yazar E. (2000). Effects of continuous supplementations of ascorbic acid, aspirin, vitamin E and selenium on some haematological parameters and serum superoxide dismutase level in broiler chickens. Br. Poult. Sci..

[31] Wang X., Lin H., Gu Y. (2012). Multiple roles of dihomo-γ-linolenic acid against proliferation diseases. Lipids Health Dis..

[32] Aviagen Ross 308 Broiler: Performance Objectives. www.avigan.com.

[33] Lerner K.G., Glick B., McDuffie F.C. (1971). Role of the bursa of fabricius in IgG and IgM production in the chicken: Evidence for the role of a non-bursal site in the development of humoral immunity. J. Immunol..

[34] Sigolo S., Deldar E., Seidavi A., Bouyeh M., Gallo A., Prandini A. (2019). Effects of dietary surpluses of methionine and lysine on growth performance, blood serum parameters, immune responses, and carcass traits of broilers. J. Appl. Anim. Res..

[35] Van Limbergen T., Sarrazin S., Chantziaras I., Dewulf J., Ducatelle R., Kyriazakis I., McMullin P., Méndez J., Niemi J.K., Papasolomontos S. (2020). Risk factors for poor health and performance in European broiler production systems. BMC Vet. Res..

[36] Golrokh A.J., Bouyeh M., Seidavi A., Hoven R.v.D., Laudadio V., Tufarelli V. (2016). Effect of different dietary levels of atorvastatin and L-carnitine on performance, carcass characteristics and plasma constitutes of broiler chickens. J. Poult. Sci..

[37] Shabani S., Seidavi A.R., Asadpour L., Corazzin M. (2015). Effects of physical form of diet and intensity and duration of feed restriction on the growth performance, blood variables, microbial flora, immunity, and carcass and organ characteristics of broiler chickens. Livest. Sci..

[38] Seidavi A., Asadpour L., Dadashbeiki M., Payan-Carreira R. (2014). Effects of dietary fish oil and green tea powder supplementation on broiler chickens immunity. Acta Sci. Vet..

[39] Pourhossein Z., Qotbi A.A.A., Seidavi A., Laudadio V., Centoducati G., Tufarelli V. (2014). Effect of different levels of dietary sweet orange (*Citrus sinensis*) peel extract on humoral immune system responses in broiler chickens. Anim. Sci. J..

[40] Dibaji S.M., Seidavi A., Asadpour L., da Silva F.M. (2014). Effect of a synbiotic on the intestinal microflora of chickens. J. Appl. Poult. Res..

[41] Jang I., Ko Y., Kang S., Lee C. (2007). Effect of a commercial essential oil on growth performance, digestive enzyme activity and intestinal microflora population in broiler chickens. Anim. Feed. Sci. Technol..

[42] Fathi M., Haydari M. (2016). Influence of dietary aspirin on growth performance, antioxidant status, and mortality due to ascites in broiler chickens. Poult. Sci. J..

[43] Stilborn H., Harris G., Bottje W., Waldroup P. (1988). Ascorbic acid and acetylsalicylic acid (aspirin) in the diet of broilers maintained under heat stress conditions. Poult. Sci..

[44] Ahmadu S., Mohammed A.A., Buhari H., Auwal A. (2016). An overview of vitamin C as an antistress in poultry. Malays. J. Vet. Res..

[45] Heidari M., Moeini M.M., Nanekarani S.H. (2013). Effect of vitamin C, acetylsalicylic, NaHCO_3_ and KCL supplementation on the performance of broiler chickens under heat stress condition. J. Agric. Technol..

[46] Kutlu H., Forbes J. (1993). Changes in growth and blood parameters in heat-stressed broiler chicks in response to dietary ascorbic acid. Livest. Prod. Sci..

[47] Takahashi K., Akiba Y., Horiguchi M. (1991). Effects of supplemental ascorbic acid on performance, organ weight and plasma cholesterol concentration in broilers treated with propylthiouracil. Br. Poult. Sci..

[48] Omidi S., Bouyeh M., Seidavi A. (2023). The effect of vitamin C and aspirin supplementation on the performance of turkeys under normal conditions. Anim. Biotechnol..

[49] Crespo N., Esteve-Garcia E. (2001). Dietary fatty acid profile modifies abdominal fat deposition in broiler chickens. Poult. Sci..

[50] Tzeng R.-Y., Becker W.A. (1981). Growth patterns of body and abdominal fat weights in male broiler chickens. Poult. Sci..

[51] Deaton J., Lott B. (1985). Age and dietary energy effect on broiler abdominal fat deposition. Poult. Sci..

[52] Tang S., Zhou S., Yin B., Xu J., Di L., Zhang J., Bao E. (2018). Heat stress-induced renal damage in poultry and the protective effects of HSP60 and HSP47. Cell Stress Chaperones.

[53] Boostani A., Ashayerizadeh A., Mahmoodian F.H., Kamalzadeh A. (2010). Comparison of the effects of several feed restriction periods to control ascites on performance, carcass characteristics and hematological indices of broiler chickens. Braz. J. Poult. Sci..

[54] Daneshyar M., Kermanshahi H., Golian A. (2009). Changes of biochemical parameters and enzyme activities in broiler chickens with cold-induced ascites. Poult. Sci..

[55] Fahmideh M.N., Seidavi A., Bouyeh M. (2023). The effect of different levels of vitamin C and chromium on growth performance, carcass characteristics, digestive organs, immunity, blood constituents, liver enzymes, cecal microflora, meat sensory taste and fatty acid profile of breast meat in broilers. Vet. Med. Sci..

[56] Kong F., Zhao G., He Z., Sun J., Wang X., Liu D., Zhu D., Liu R., Wen J. (2021). Serum creatine kinase as a biomarker to predict wooden breast in vivo for chicken breeding. Front. Physiol..

[57] Mohan K., Jayakumar K., Narayanaswamy H., Pavithra B., Bayer-Darmel M. (2012). Study of potential toxic effects of acetylsalicylic acid upon short-term repeated oral administration in chickens. J. Appl. Anim. Res..

[58] Shi X., Ding M., Dong Z., Chen F., Ye J., Wang S., Leonard S.S., Castronova V., Vallyathan V. (1999). Antioxidant properties of aspirin: Characterization of the ability of aspirin to inhibit silica-induced lipid peroxidation, DNA damage, NF-κB activation, and TNF-α production. Mol. Cell. Biochem..

[59] Rajani J., Torshizi M.K., Rahimi S. (2011). Control of ascites mortality and improved performance and meat shelf-life in broilers using feed adjuncts with presumed antioxidant activity. Anim. Feed. Sci. Technol..

[60] Moghaddam A.Z., Hassanpour H., Mokhtari A. (2009). Oral supplementation with vitamin C improves intestinal mucosa morphology in the pulmonary hypertensive broiler chicken. Br. Poult. Sci..

[61] Amer S.A., Mohamed W.A.M., Gharib H.S.A., Al-Gabri N.A., Gouda A., Elabbasy M.T., El-Rahman G.I.A., Omar A.E. (2021). Changes in the growth, ileal digestibility, intestinal histology, behavior, fatty acid composition of the breast muscles, and blood biochemical parameters of broiler chickens by dietary inclusion of safflower oil and vitamin C. BMC Vet. Res..

[62] Konieczka P., Barszcz M., Kowalczyk P., Szlis M., Jankowski J. (2019). The potential of acetylsalicylic acid and vitamin E in modulating inflammatory cascades in chickens under lipopolysaccharide-induced inflammation. Vet. Res..

[63] Lohakare J.D., Kim J.K., Ryu M.H., Hahn T.-W., Chae B.J. (2005). Effects of vitamin C and vitamin D interaction on the performance, immunity, and bone characteristics of commercial broilers. J. Appl. Poult. Res..

[64] Abdulameer Y.S. (2019). The effects of dietary vitamin C and Citrus Sinensis peel on growth, hematological characteristics, immune competence, and carcass characteristics in broilers exposed to heat stress. Iraqi J. Vet. Sci..

[65] Sheikh I.S., Bajwa M.A., Rashid N., Mustafa M.Z., Tariq M.M., Rafeeq M., Samad A., Asmat T.M., Ullah A. (2020). Effects of immune modulators on the immune status of broiler chickens. Pak. J. Zool..

[66] Shojadoost B., Yitbarek A., Alizadeh M., Kulkarni R.R., Astill J., Boodhoo N., Sharif S. (2021). Centennial review: Effects of vitamins A, D, E, and C on the chicken immune system. Poult. Sci..

[67] Carr A.C., Maggini S. (2017). Vitamin C and immune function. Nutrients.

[68] Jafari D., Esmaeilzadeh A., Mohammadi-Kordkhayli M., Rezaei N. (2019). Vitamin C and the immune system. Nutrition and Immunity.

[69] Watson R., Zibadi S., Preedy V. (2010). Dietary Components and Immune Function.

[70] Hussain M., Javeed A., Ashraf M., Zhao Y., Mukhtar M.M., Rehman M.U. (2012). Aspirin and immune system. Int. Immunopharmacol..

[71] Dieter M.P., Breitenbach R. (1968). The growth of chicken lymphoid organs, testes, and adrenals in relation to the oxidation state and concentration of adrenal and lymphoid organ vitamin C. Poult. Sci..

